# Antivaccine Movement and COVID-19 Negationism: A Content Analysis of Spanish-Written Messages on Twitter

**DOI:** 10.3390/vaccines9060656

**Published:** 2021-06-15

**Authors:** Ivan Herrera-Peco, Beatriz Jiménez-Gómez, Carlos Santiago Romero Magdalena, Juan José Deudero, María García-Puente, Elvira Benítez De Gracia, Carlos Ruiz Núñez

**Affiliations:** 1 Nursing Department, Faculty of Medicine, Alfonso X El Sabio University, 28691 Villanueva de la Cañada, 28040 Madrid, Spain; bgomejim@uax.es; 2 Faculty of Health Sciences, Alfonso X el Sabio University, Avda Universidad, 1, 28691 Villanueva de la Cañada, 28040 Madrid, Spain; cromemag@uax.es (C.S.R.M.); jpenadeu@uax.es (J.J.D.); eben@uax.es (E.B.D.G.); 3 Fdn Jiménez Díaz, Avda. Reyes Católicos, 2, AlterBiblio, 28040 Madrid, Spain; maria@alterbiblio.com; 4 Loja High Resolution Hospital, APS Poniente, Av. Tierno Galván, Loja, 18300 Granada, Spain; cruiznu@gmail.com

**Keywords:** COVID-19, fake news, misinformation, public health, Twitter, vaccines

## Abstract

During the COVID-19 pandemic, different conspiracies have risen, with the most dangerous being those focusing on vaccines. Today, there exists a social media movement focused on destroying the credibility of vaccines and trying to convince people to ignore the advice of governments and health organizations on vaccination. Our aim was to analyze a COVID-19 antivaccination message campaign on Twitter that uses Spanish as the main language, to find the key elements in their communication strategy. Twitter data were retrieved from 14 to 28 December using NodeXL software. We analyzed tweets in Spanish, focusing on influential users, most influential tweets, and content analysis of tweets. The results revealed ordinary citizens who ‘offer the truth’ as the most important profile in this network. The content analysis showed antivaccine tweets (31.05%) as the most frequent. The analysis of anti-COVID19 tweets showed that attacks against vaccine safety were the most important (79.87%) but we detected a new kind of message presenting the vaccine as a means of manipulating the human genetic code (8.1%). We concluded that the antivaccine movement and its tenets have great influence in the COVID-19 negationist movement. We observed a new topic in COVID-19 vaccine hoaxes that must be considered in our fight against misinformation.

## 1. Introduction

The COVID-19 disease, which started in Wuhan, China, had its first case reported in December 2019 and continues today [1,2]. COVID-19 was defined by the World Health Organization (WHO) as a public health emergency and declared a pandemic on 11 March 2020 [3], and it has led to multiple negative effects on health and economy globally [4]. The negative impact on public health, as well as the lack of treatment and prevention measures (such as vaccines) [5], led many countries to develop both drugs and vaccines to fight SARS-CoV-2 [6]. 

Although vaccines have historically been one of the most relevant successes in terms of public health due to their key role the prevention of infectious diseases in human populations [7], they have had to face many detractors such as groups of people who are against vaccination [8]. These groups are defined as the antivaccine movement, which denies the advantages and benefits of vaccines under the premise of being exposed to a greater risk based on false information about vaccine security, composition, or even side-effects [9].

Just 1 year after the SARS-CoV-2 coronavirus outbreak that caused the pandemic, the scientific community achieved, after great efforts, effective vaccines against COVID-19 [10], which should be enough to increase the positive opinion about vaccines in order to achieve herd immunity through voluntary vaccination. However, one of the greatest difficulties in achieving this scenario is related to the high volume of misleading and manipulated information, lacking scientific evidence, which quickly spreads on the Internet in general and on social networks in particular [11,12].

The arguments used against vaccines are manipulated and even invented [13]. For example, these groups spread messages with out-of-context, invented, or exaggerated information about a vaccine’s adverse effects, using the most widespread social networks worldwide: Twitter, YouTube, Instagram, and Facebook, among others [14,15,16]. This means that the information can reach a large number of people, spreading disinformation [17], even generating fear [18], and leading to the perception that vaccines are a threat [19]. This is why these kinds of messages can be considered a public health threat [20]. In addition, we have to take into account so-called health anxiety disorder, as it is linked to a great amount of incorrect and ambiguous information to which people have access to [21,22]. This can generate abnormal behaviors both at individual and social levels, due to the loss of trust in health authorities, the blaming of people who belong to different ethnic, social, or political groups, and the development of conspiratorial thoughts [23].

Regarding media usage, the Internet is the main platform for antivaccine supporters to spread their ideas through blogs, webpages [24], and social network messages. We cannot ignore the use of communication technologies (empathic media), which have the capacity to evaluate and analyze human feelings expressed on social networks and use them to manipulate public opinion [25].

Today, during the COVID-19 pandemic, the antivaccine movement has made social networking its best tool for spreading false or biased information [26]. Unlike the static characteristics of websites, social networks are designed to quickly spread information and encourage fast-paced dialogues and interactions between users [25,26,27]. An analysis of the antivaccine discourse on social networks pointed out that “the lines of argument focus on safety, effectiveness, importance, and people’s values and beliefs” [28]. The importance of source and message analysis is becoming increasingly necessary in order to fight against the social media-generated infodemic associated with the COVID-19 pandemic [29].

The use of social networks as new vehicles for the spread and dispersion of information is undeniable in modern society. Among these social networks, Twitter is one of the most widespread and popular, globally. On the other hand, it is worth noting that Spanish is currently the third most used language on the Internet and the second most frequent language on Twitter, in terms of both users and published messages [30]. This means that the Spanish-speaking community generates and consumes a lot of information [31]. Although arguments against COVID-19 vaccines use the same strategies as in other platforms (presenting false facts, unfounded arguments, distorted scientific evidence, etc.), on Twitter, we find new formulas, since the character limit does not allow to contextualize the information, making it the perfect breeding ground for the spread of sensationalist false facts or manipulated data [27]. In addition, the antivaccine movement has gained 7.8 million supporters; thus, it is foreseeable that this movement could undermine the launch of any therapeutic option against COVID-19 [32].

This study aimed to analyze the COVID-19 antivaccination message campaign so that it can serve to develop recommendations for public health authorities in order to fight against this kind of discourse that threatens public health. We should not forget that vaccines represent the best option to achieve large-scale immunity [33], whereas the spread of false information may sabotage our efforts against the pandemic [34,35]. Furthermore, if disinformation and misinformation about vaccines continue to be spread by antivaccine groups using this communication strategy on social media, it is possible to predict that antivaccination supporters will gain a dominant discourse on social media around 10 years from now [36].

According to the above, our main objective was to (i) analyze the hashtag #yonomevacuno (#idonotgetvaccinated) as a dangerous movement to public health in the current pandemic, as well as influential users within the #yonomevacuno movement, (ii) determine the level of interactions between user groups in the network #yonomevacuno, (iii) analyze the presence and role of official institutions in the #yonomevacuno movement in blocking antivaccine messages, and (iv) analyze the messages (tweets) and their content, sent on this network. Lastly, we consider that this study may have practical value to develop recommendations for public health authorities.

## 2. Materials and Methods

### 2.1. Study Design and Ethics

An observational, retrospective, and time-limited study is proposed, in which activity on the social network Twitter was analyzed.

This study was considered exempt from ethical review because it was performed on a social network, and it did not interfere with any patient or human data beyond measuring Internet activity among Twitter users. Moreover, this study only used data from users who consented on Twitter to disclose their data publicly (i.e., no privacy settings were selected by them). However, accounts of individual users were anonymized in order to develop good research practices on social networks [36].

### 2.2. Data Collection

The information from tweets was extracted through an API (application programming interface) search tool, using the professional version of the software NodeXL (Social Media Research Foundation).

To achieve the objectives proposed in this study, the keyword “yonomevacuno” (idonotgetvaccinated) and the hashtag #yonomevacuno were selected, taking into account the situation created after the beginning of the worldwide vaccination campaigns.

The Twitter users included in the data analysis were those who sent tweets with the abovementioned characteristics during a predefined period. Unverified users were also included, as one of the objectives of the study was to analyze message dissemination.

The tweet selection criteria for this study were (i) tweets published in the Spanish language, (ii) tweets containing the hashtag #yonomevacuno, the keyword “yonomevacuno”, or the phrase “yo no me vacuno”, and (iii) tweets that were published between 14 December (12:00 a.m. CET) and 28 December 2020 (11:59 p.m. CET). The time frame selected for this study is related to the date that the European Medicines Agency (EMA) announced the authorization to start vaccination against COVID-19 with Pfizer BionTech’s vaccine (21 December 2020). We collected data from the activity of users on Twitter within the network ‘yonomevacuno’ 7 days before and after the day of announcement, with the aim of obtaining an understanding of the activity of ‘yonomevacuno’ supporters before and after the aforementioned date.

With the data collected from the hashtag #yonomevacuno, it was observed that a total of 5040 Twitter users participated, generating a total of 1,664,261 impressions. In addition, it was found that there was a total of 12,340 interactions classified as follows: 1724 tweets (13.98%), 6828 retweets (55.33%), 2179 mentions (17.66%), and 1608 replies (13.03%).

### 2.3. Data Analysis

The analysis of the data obtained was performed in several steps. The first step was to analyze the most influential Twitter users who posted under the aforementioned hashtag, as well as their characteristics. We used a traditional social network analysis technique, the betweenness centrality score (BCS). This centrality measure, in social network terms, is associated with the user’s power within the network, denoting the importance of connecting and transmitting information across the entire network [37]. BCS measures the influence of a vertex over the flow of information to other vertices, always assuming that information will travel through the shortest vertex path. The BSC value reflects how a user can control the information, choosing whether to share it or not, disclosing it to their network [38,39]. In our study, BCS was the value used to define influential users in the #yonomevacuno network. The Twitter users were compiled and grouped by nodes using the Clauset–Newman–Moore cluster algorithm [40].

The hypothetical “yonomevacuno” network activity allowed us to identify the content, activities, and/or influential users that would be strongly associated with overall Twitter activity, measured by the metrics of total tweets, impressions, retweets, and replies [41]. It is important to define that tweets entail the creation of original content by the user, whereas retweets are an indicator that shows the transmission of a tweet sent by another user (it is not original content). Lastly, the impression is an indicator of the propagation of information, obtained when the number of tweets is multiplied by the number of followers [41].

Secondly, a graphical network interaction analysis was carried out. Lastly, a content analysis was performed with the categories created after analyzing the data. It is important to note that, in this category analysis, only original tweets were taken into account, since these were considered to be those that generated the actual content disseminated throughout the user network. The content and category coding was performed independently by two researchers and corroborated by a third person, whereby any differences in approach and focus were always discussed and resolved with full agreement.

## 3. Results

### 3.1. Social Network Analysis

**Figure 1:**
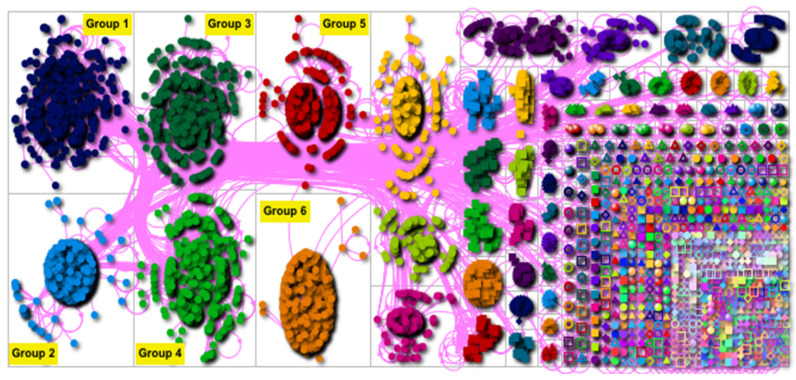
Social media network connections in #yonomevacuno. Graphical representation of the importance of controlling information of clusters of users, where only the six most important groups are indicated on the basis of their BCS value. Group 1 is mostly composed of users geolocated in Argentina; Groups 2 and 4 are mostly made up by users in Spain; Group 3 mainly involves users in Spain and Mexico; lastly, Group 4 is mostly composed of users in Colombia.

Figure 1 shows the analysis performed on the social network around the hashtag #yonomevacuno, in which all users and communities who shared this hashtag are represented, regardless of the content of their tweets.

When observing Figure 1, we can determine the existence of six main groups, which, after being analyzed, can be defined as spreading information groups. Nevertheless, each group has its own characteristics in terms of messages and geolocation of users.

In addition, all groups were found to have a similar size, representing 66.14% of #yonomevacuno network users, whose impact oscillated from 595 users in Group 1 (11.8%) to 511 users in Group 6 (10.14%).

In relation to the messages sent by each group, we can see that all groups were characterized by being focused on the dissemination of information including the hashtag #yonomevacuno. Table 1 shows frequent examples of the types of tweets in each group. Group 1 is characterized by tweets associated with criticizing politicians and/or government, as well as those including antivaccine information. In Group 2, the messages are focused on the dissemination of information about the anti-COVID-19 vaccine and conspiracy theories (5G and chips in vaccines). In Group 3, most of the tweets refer to anti-COVID-19 vaccine (vaccine safety) messages and COVID-19 denial. On the other hand, the most common messages in Groups 4 and 5 are framed within the category of conspiracy theories (genocide), COVID-19 denial, and anti-COVID-19 vaccine. The most common messages within Group 6 are related to the categories of reluctant but open-minded tweets and criticizing politicians and/or government.

Lastly, the information related to user geolocation was evaluated, since the hashtag #yonomevacuno is used internationally. Users with authenticated geolocation were assessed, and the following results were obtained: (i) in Group 1, most of the users with verified geolocation (40%) were in Argentina; (ii) in Group 2, it was found that, among the users with verified geolocation (22.79%), most of them were based in Spain; (iii) in Group 3, the users with verified geolocation (22.76%) were based in Spain and Mexico; (iv) most of the verified geolocation users in Group 4 were located in Chile (41.35%); (v) in Group 5, most of the verified users (30.61%) were located in Spain; (vi) Group 6 was formed by users located mainly in Colombia (38.75%).

### 3.2. User Analysis

The 10 most influential Twitter users fitting in the above categories were collected, using the value of interaction as a measure of importance in the network analyzed (Table 2). A general description of the account is provided so it can be identified. Additionally, the value “pagerank” is provided, which measures the possibility that the messages in that account are reached by any given user on Twitter (with a probability of 85%). The number of followers of these accounts during the analyzed time window is also provided, including only those users who had more than 1000 followers. Lastly, information about the group in which they are included, as defined in Figure 1, is provided.

Among the users appearing on Table 2, we can observe that User 1 was an anonymous account with a profile openly focused on the dissemination of conspiracy theories related to COVID-19 and vaccines. User 1 was the most popular account. It was an active user, with 57 tweets published that generated 78,968 interactions among users, representing 4.75% of the generated information traffic. Furthermore, User 1 also participated by sending multiple messages that could be framed within the conspiracy category (Table 2). On the other hand, it was observed that, for some accounts (Users 8, 9, and 10), most of the messages were focused on sharing information criticizing COVID-19 vaccines. Lastly, it was found that the account defined as User 10 was the most active account in the network #yonomevacuno, with 150 tweets published.

It is worth noting that the Ministry of Health of Colombia was also registered as one of the most relevant accounts according to BCS, despite not having created any tweet with the hashtag #yonomevacuno.

### 3.3. Content Analysis

The entire sample of tweet interactions collected (*n* = 1591) within the hashtag #yonomevacuno was analyzed, and it was found that 942 users generated that number of tweets, suggesting an average of 1.65 tweets per user.

Considering the different approaches within the #yonomevacuno movement, all the published tweets under this hashtag were analyzed in order to categorize them (Table 2), where we can see a predominance of certain tendencies. The anti-COVID-19 vaccine stream accounted for a total of 31.05% of the tweets in the network #yonomevacuno; it was followed by the group of tweets that did not express any specific opinion (28.85%), with conspiracy theory tweets (16.97%) being the third main tendency.

Within the hashtag #yonomevacuno, it is worth mentioning the existence of a series of tweets from users in favor of the COVID-19 vaccine (4.15%), including those published by an account located in Spain, the author of which is presented as a pharmacist, focused on dismantling the vaccine hoax related to the COVID-19 vaccine.

Furthermore, we observed tweets from users defined as reluctant to vaccinate but with open-minded behavior (1.95%). The content of these messages was related to the expression of an idea/desire to be vaccinated as long as the vaccines meet all of the safety standards required by international organizations. These kinds of users were located in Latin American countries, where the Sputnik-V vaccine would be distributed.

In relation to the antivaccine messages, it is important to note that 494 tweets were generated from the 1591 sample tweets. The tweets categorized as such used arguments focused on indicating that (i) the vaccines are not safe (63.36% of tweets), (ii) the vaccine effectiveness is questionable (8.9%), and (iii) vaccines are business (8.7%), as well as (iv) tweets which divulged unverified information framed as beliefs about the effect and even the production and transport of the vaccines (18.83%) (see Table 3).

### 3.4. Anti-COVID-19 Vaccine Content Analylsis

One of the main components of the #yonomevacuno network discourse can be included within antivaccine messages. In order to exactly identify the types of messages, an analysis of messages was performed.

We observed that messages listed in the antivaccine category involved content questioning vaccine safety. Within this group, 79.87% of the messages indicated the existence of adverse effects derived from the use of COVID-19 vaccines, whether from Pfizer, Moderna, or Sputnik. The last group of messages (7.03%) was focused on accusing the COVID19 vaccine of being responsible for the emergence of diseases, focusing on the outbreak of the new strain of the SARS-CoV-2 virus that appeared in the UK in December 2020.

In the vaccine efficacy category, it was observed that 54.4% of the messages indicated that the COVID-19 vaccine was ineffective, while 36.6% of the messages suggested that the COVID-19 vaccine did not work as assured by the health authorities or pharmacy companies. Lastly, 9.09% of the messages insisted on spreading information about the existence of more effective treatments than the vaccine, such as Miracle Mineral Solution (MMS), a sodium chlorite product (Table 4).

Regarding the vaccine importance category, 44.19% of the messages insisted on the necessity of acquiring natural immunity from COVID-19 infection rather than being immunized by the vaccine. Furthermore 55.81% of the messages emphasized that the vaccination strategy was created by governments so the pharmaceutical industry could economically benefit from it, while adding that the vaccine is not necessary (Table 4).

Within the beliefs about COVID-19 vaccine category, we could find messages expressing a certain religious content in which it was emphasized that God would protect against COVID-19 (9.68%). Moreover, 5.68% of the messages included information claiming that natural alternatives, such as sunbathing, healthy eating, and daily physical exercise, were better immunity options than the vaccine. It was also found that 8.6% of the messages insisted on the hypothesis that vaccines are a fraud created by the pharmaceutical industry. On the other hand, 76.34% of the messages included in the category of misinformation about how vaccines work (Table 4) were focused on information about how vaccines work, as well as their composition and logistic details.

The last group analyzed (Table 3) accounted for 8.1% of the messages classified as antivaccine and was associated with the dissemination of information related to the hypothesis that anti-COVID-19 vaccines would serve to manipulate the human genetic code (Table 4).

Furthermore, Table 5 shows an analysis of the external links that served to assess the types of documents used to reinforce these messages.

The first URL notes that the Vatican endorses the use of vaccines using cell lines. This argument has been used by antivaccine users when the reality is that, in the same news item, it is stated that this hypothesis is not true. The second URL leads to a piece of news in a Spanish newspaper indicating that a new treatment against COVID-19 is being investigated. This URL can be found in tweets claiming that the vaccine is not necessary. The third URL leads to a site that promotes and defends the freedom of vaccination by providing legal information on how to avoid vaccination, as well as information on the alleged harmful effects of vaccination. The fourth URL leads to an online newspaper that shows information about the implementation of a vaccination certificate to register people vaccinated against COVID-19 in Spain. The fifth URL provides access to a video in which 33 alleged health professionals appear talking against vaccination. However, after analyzing the video, we can see that many of them are neither health professionals nor active workers and, in addition, many of them are linked to the antivaccine movement. The sixth URL explains the arrival of the Sputnik-V vaccine in Argentina, and the seventh URL explains how the number of people reluctant to be vaccinated in Spain has decreased. The eighth and ninth URLs are associated with digital media. The former informs about the absence of legal repercussions for pharmaceuticals regarding vaccine adverse effects, and the latter explains that the World Health Organization has modified the definition of herd immunity to adapt it to the current situation.

Lastly, there is a link to a newspaper article in which it is stated that a Spanish surgeon warned of the appearance of encephalitis as an adverse effect of the vaccine. This health professional is actually a doctor and is currently practicing as such.

## 4. Discussion

The present study focused on message analysis within the network #yonomevacuno. These messages were written in Spanish via Twitter, and all of them included information about the vaccine, vaccination process, or the COVID-19 pandemic, where users expressed a diversity of opinions about the management of the pandemic.

In our study, we observed the emergence of six opinion groups of users that made up 66.14% of the network’s user community. Moreover, these groups were clearly different from each other, and, in some cases, they were very well defined in terms of the type of messages sent. The groups can be also defined in terms of country geolocation, as the users claimed to be from countries such as Argentina, Chile, Colombia, or Spain, among others.

This demonstrates that the #yonomevacuno campaign is neither hierarchical nor dominated by a single user group that disseminates all the information; rather, there are multiple groups that have a similar impact when disseminating messages. It is also observed that there is not a high amount of interaction between the groups, which could be explained by the different typology of messages found in the network and the countries where the users are located. These data coincide with the literature where these kinds of users or activists even create a disproportionate social media footprint, due to an effective online-communication strategy [35,42], but they are small and loosely organized [43,44]

All these data seem to indicate that there are numerous users who disseminate messages within the “yonomevacuno” movement. The information spread is not centralized, showing that this initiative arises in a plural and disseminated way across different countries, communities, and people who oppose the vaccine, whether for ethical, moral, or political convictions, or because they are simply misinformed. According to our results, as with other conspiracy theories spread on Twitter associated with the COVID-19 pandemic, the misinformation and fear message against COVID-19 vaccination could have been minimized if the main accounts were quickly identified and blocked [3].

Regarding our second working hypothesis, it was observed that the Colombian Ministry of Health appears among the most relevant accounts in the dissemination of this information [3]. However, this account has not actively participated in the conversation generated around the #yonomevacuno network; thus, it does not produce verified content that could serve as an element to combat the generated misinformation. In addition, this account was often quoted in a Colombia-focused group, in which the messages mostly focused on political criticism rather than falling into other #yonomevacuno categories. When analyzing the users, we found that there is no public organization, within the network #yonomevacuno, with the aim of generating verified content or offering counter-information against the #yonomevacuno discourse. This type of situation has been described previously in other social media campaigns, such as the influenza vaccine campaign in Spain [43] or campaigns like the World Mental Health Awareness Day [44], where the public institutions had little presence and the “fight” against the discourse was led by particular users [38,39], which can be considered as one of the key elements to explain the absence of other sources of information to counteract the misinformation [17,45,46,47,48] about the pandemic and vaccines. This situation generates fear [18], uncertainty [47], and the perception that vaccines are a threat to health [19].

Lastly, we proceeded to analyze all tweets, since they are the items through which information is subsequently disseminated within the network. It is worth noting that most tweets (31.05%) included antivaccine content. However, the presence of tweets with content unrelated to the #yonomevacuno narrative suggest that there are many users who do not support the movement. The group sharing conspiracy theories ranked third in terms of presence. This evidence supports the thesis that there are situations in which misinformation can lead to the development of conspiratorial thoughts and ideas [17,21,49]. Furthermore, it is so important to understand that antivaccination users offer a wide range of potentially attractive narratives that blend topics such as safety concerns, conspiracy theories, and the cause and cure of COVID-19, amongst others [35,36,46,47].

On the other hand, it was found that many users under the #yonomevacuno movement shared the tendency to criticize politicians and/or governments with a different political ideology than their own. These users generated messages of distrust toward public bodies, which they blamed for the current situation [18].

Regarding our last research objective, where the goal was to analyze tweets sent to the network #yonomevacuno, we observed that the main group involved users expressing a defined opinion or idea related to antivaccine content (Table 3). It was observed that the categories found were in line with the most common postulates of antivaccine groups [47,50], with the message adapted to the COVID-19 vaccine in this case [28].

When analyzing antivaccine messages, it was observed that there was a predominance of messages strongly focused on spreading fear, which is in line with the findings of other authors [3,18,19]. It should also be noted that a high percentage of antivaccine tweets were focused on disseminating misrepresented information, either through ignorance or intentionally, associated with vaccine components, as well as their production or transportation [8,45]. In our study, there was a predominance of messages questioning vaccine safety, mainly through an overestimation of the adverse effects of anti-COVID-19 vaccines, even sharing false information regarding death cases due to such adverse effects. Accordingly, being one of the most interesting findings within the dissemination of messages in the antivaccine collective, there was a typology of messages sharing the false information that mRNA vaccines against COVID-19 would produce changes in human DNA, indicating that human beings would no longer be able to consider themselves human, instead turning into genetically modified organisms. These messages were shared along with more alarmist content, in which it was claimed, in an unfounded manner and based on personal opinions and beliefs, that the vaccine against COVID-19 [48] was responsible for the mutation of the virus and for the new strain which was discovered in the UK in December 2020 [50].

Likewise, when analyzing the most shared URLs within the antivaccine messages, we found a common argument that denounces the use of aborted fetuses in the production of vaccines. This theory is totally false and can be associated with religious beliefs and antiabortion movements. There was also a noticeable presence of information regarding alleged health professionals who spread fear and uncertainty about vaccine and vaccination strategies or even messages distorting statements pronounced by health professionals in order to match them with false ideas about vaccine safety and adverse effects.

It could also be observed that the messages from users disseminating antivaccine messages are in line with the discourse aimed at generating fear and uncertainty about vaccines [19]. This is the discourse that antivaccine collectives have historically used [9]. However, in some cases, the information was adapted to fit it into the characteristics of the development and technologies associated with anti-COVID-19 vaccines.

Lastly, it should be noted that this study had a number of limitations, such as using only Twitter; thus, the user campaign analysis was limited to this social network. Furthermore, retrieving information using a specific hashtag and keyword may have missed users who posted messages against COVID-19 vaccination without using these keywords. Another limitation, which will require future analysis, is related to whether the users included in this network were bots or real users, in order to analyze the dispersion of information from both types of accounts.

There have been previous studies discussing the importance of developing strategies to counteract the antivaccine discourse, as a strategy to avoid problems in the acceptance and demand for vaccination against COVID-19 [51,52]. However, we want to highlight an important strength of this study, which, to the knowledge of the authors, is the first to address an analysis of the behavior of antivaccine supporters and negationists, i.e., Twitter users who deny the necessity and the effectiveness of vaccines against COVID-19 as a public health strategy.

## 5. Conclusions

To the knowledge of the authors, the present study is the first to address the analysis of a network focused on the dissemination of COVID-19 antivaccination messages in the Spanish-speaking community on Twitter.

We believe that the results shown in this study provide insight into the typology of antivaccine messages, as well as a way to identify user accounts disseminating antivaccination messages that may boycott the efforts of health organizations to achieve a high vaccination rate as a preventive measure against the pandemic.

It is essential to develop and implement public health surveillance programs that include the monitoring of social networks as a priority action. The creation of “observatories” that assess conversations in social networks on public health issues will allow a rapid response against misinformation; the longer this takes is the more complex it will be to get the right information to the population. However, these actions must be led by healthcare organizations, both public and private.

Surveillance programs should also include communication actions focused on creating and disseminating verified health content in appropriate and understandable formats for the population to counteract the misinformation generated. From our point of view, it is necessary that healthcare organizations are present in potentially hazardous hashtags, to offer correct discourse and a reliable source of health information. Although it is extremely important for public institutions to be present and lead these campaigns, we believe it to be very important that these institutions do not prompt rejection of certain parts of the population; this is where healthcare professionals, as well as individual users, can act as reference figures to which users of social networks can turn to, in order to obtain healthcare information and, of course, combat healthcare misinformation and disinformation.

We believe that this situation suggests the need to implement training actions for healthcare professionals and even citizens in the use of social networks to enhance their participation and improve the effectiveness of communication. It is important to focus these actions on showing how to prepare reliable tweets, focusing on the veracity of the content, attaching external and reliable sources, and taking care when writing the tweets. Furthermore, these users should be trained in communication techniques with two purposes: (i) to avoid making jokes about the (lack of) scientific knowledge of users or even their beliefs; (ii) to avoid conflict, insults, or aggressive actions against antivaccine supporters or COVID-19 negationists. The main procedure of action should simply involve explaining why or how the vaccines do what they suggest they do.

## Figures and Tables

**Table 1:** Most interacted tweets among groups described in Figure 1.

Tweet	Group from NodeXl	Interactions
Caution! Vaccination was suspended in Rosario because ALL vaccinated people are having vomiting and fever. #YoNoMeVacuno (#IDoNotGetVaccinated) #NoEsElCovidEsElTotalitarismo (#ItIsNotCovidItIsTotalitarism)	1	5446
Having chips in vaccines is a crackpot thing, right? It is not But don’t worry, it is just a barcode to check your lot and expiration date #COVID19 #Plandemia #YoNoMeVacuno (#Plandemic #IDoNotGetVaccinated)	2	350
My body, MY CHOICE #YoNoMeVacuno (#IDoNotGetVaccinated)	3	1151
Vaccine does not guarantee anything!! #YoMeVacuno #YoNoMeVacuno (#IDoGetVaccinated #IDoNotGetVaccinated)	4	124
Pedro Cavadas | Encephalitis: the “adverse effect” of the “quick” anti-coronavirus vaccines noted by Dr. Cavadas. This is the only EXPERT I trust… #YoNoMeVacuno (#IDoNotGetVaccinated)	5	1216
#YoNoMeVacuno (#IDoNotGetVaccinated), but not because I do not want to. I live in Colombia. Here, there are no vaccines. Our president is rehearsing for his reality show.	6	1748

**Table 2:** Influential users ranked according to their betweenness centrality score (BCS).

Rank	Account Description	BCS	Pagerank	Followers	Group
User1	Anonymous profile focused on offering the truth.	12,047,282.99	218.69	7446	G2
User2	User defined as writer and senator in Colombia	5,590,037.244	232.249	1,004,405	G6
User3	Citizen focused on political activism	4,022,155.431	143.997	2676	G7
User4	Citizen defined as feminist	3,818,752.493	20.593	3787	G10
User5	Citizen defined as writer	3,722,722.425	123.186	11,292	G5
User6	Official account of Colombia Ministry of Health)	3,317,916.869	1.416	1,395,041	G4
User7	User defined as a freethinker	227,136.274	8.388	6297	G10
User8	Anonymous profile	2,191,083.218	55.656	1183	G3
User9	Citizen defined as engineer	2,089,182.493	0.878	2206	G10
User10	Anonymous profile focused on political activism	2,085,652.241	44.616	15,344	G1

**Table 3:** Content analysis of individual tweets from #yonomevacuno.

Category	Sub-Category	Tweets vs. Total Tweets (*N*, %)	Tweets vs. Category Tweets (*N*, %)
Conspiracy theory tweets		270 (16.97)	
	5G and chip inoculation	14 (5.18)	
	Genocide	78 (28.89)	
	New World Order and control of population	178 (65.93)	
Criticizing politicians and/or government		218 (13.7)	
Deniers of COVID-19		53 (3.33)	
Reluctant but open-minded tweets		31 (1.95)	
Antivaccine tweets		494 (31.05)	
	Vaccine safety	313 (63.36)	
	Vaccine efficacy	44 (8.9)	
	Vaccine importance	43 (8.7)	
	Beliefs about COVID-19 vaccine	93 (18.83)	
Pro-COVID-19 vaccine tweets		66 (4.15)	
General tweets not expressing a view or opinion		459 (28.85)	

**Table 4:** Categories of anti-COVID-19 vaccine tweets and more representative tweets (most retweeted). (Note: The tweets have been transcribed verbatim).

Category	*N* (%)	Most Representative Tweets
Vaccines safety (272 tweets)		
Adverse effects	250 (79.87)	As an infectiologist, it is my civic duty to warn the population about the risk of taking an untested vaccine. The government will be responsible for a criminal, genocidal action. I wwill not hesitate to sue our nation if this happens. That is why #YoNoMeVacuno (#IDoNotGetVaccinated)
New strain of SARS-CoV-2	22 (7.03)	It is no coincidence that the UK started “vaccination” 13 days ago, and today they are announcing a new strain? Watch out! #YoNoMeVacuno (#IDoNotGetVaccinated)
Vaccine efficacy (44 tweets)		
COVID-19 vaccine is ineffective	24 (54.55)	If the vaccine does not stop you from getting #COVID19 or transmitting it, then what the fuck is it good for? Why do they insist that we get vaccinated? #Plandemia (#Plandemic) #NWO #YoNoMeVacuno (#IDoNotGetVaccinated)
COVID-19 vaccine could work or does not	16 (36.36)	Then what the fuck is the point of this vaccine?????!!! #YoNoMeVacuno (#IDoNotGetVaccinated)
There are more effective methods	4 (9.09)	The FDA and WHO lied about #dioxidodechlorine by spreading falsehoods: that it was toxic, that it was a poison, that people could die… and thousands and thousands of people have been cured but, as it is a free patent, it is not sellable. That is why I do not believe in the vaccine. #holachilelared (#hellochilenetwork) #YoNoMeVacuno (#IDoNotGetVaccinated)
Vaccine Importance (43 tweets)		
It is better to be COVID-19-positive and acquire natural immunity	19 (44.19)	I have never had a flu shot in my fucking life. I get it every year, pass it, and drop it. It is called the immune system, so how about we let it work like it has been doing for hundreds of thousands of years? #YoNoMeVacuno (#IDoNotGetVaccinated)
Government and pharmaceutical industries are allies	24 (55.81)	How nice of the pharmaceutical companies to let us buy cheap vaccines so that our governments can buy them (with our money) while the treatments for real and serious diseases (cancer, leukemia…) are priced for the rich. #YoNoMeVacuno #Plandemia #COVID19 (#IDoNotGetVaccinated #Plandemic)
Beliefs about COVID-19 vaccine (93 tweets)		
Misinformation about vaccines	71 (76.34)	The Vatican endorses vaccines using cell lines from aborted fetuses #AbortoEsGenocidio #Asesinos #YoNoMeVacuno (#AbortionIsGenocide #Murderers #IDoNotGetVaccinated).
Natural/divine alternatives	14 (15.05)	…Meanwhile, in Tel Aviv (Israel) they manage to cure 99.9% of COVID pathogens in 30 s by simply using an ultraviolet light. #YoNoMeVacuno (#IDoNotGetVaccinated)
Vaccines are a fraud from pharmaceutical industries	8 (8.6)	#YoNoMeVacuno (#IDoNotGetVaccinated) because they use you as a guinea pig. It makes no sense to apply a vaccine when you are advised to continue using a mask and social distance because the vaccine does not fully immunize you. Then why should I get the shot? This is a business from laboratories and governments.
Gene Manipulation (40 tweets)		
DNA alterations		#YoNoMeVacuno (#IDoNotGetVaccinated) because I am free and responsible for my own body, because I love myself and I refuse to be genetically manipulated or poisoned. Wake up! Let us get this over with (46 retweets)

**Table 5:** Most shared URLs within antivaccine category.

Rank	Title	URL	Times Shared
1	The Vatican endorses vaccines using cell lines from aborted fetuses	https://www.lavanguardia.com/vida/20201221/6138860/vaticano-avala-vacunas-utilizan-lineas-celulares-fetos-abortos.html (accessed on 7 January 2021)	117
2	UK scientists test new drug that guarantees immediate immunity	https://www.elmundo.es/ciencia-y-salud/salud/2020/12/26/5fe724effc6c837e3c8b4572.html (accessed on 7 January 2021)	114
3	Free vaccination	https://www.librevacunacion.com.ar/ (accessed on 7 January 2021)	62
4	Spaniards will have a vaccination record after COVID-19 vaccination	https://www.abc.es/sociedad/abci-espanoles-contaran-cartilla-vacunacion-tras-vacunarse-contra-covid-19-202012211211_noticia.html#vca=amp-rrss-inducido&vmc=abc-es&vso=tw&vli=noticia.foto (accessed on 7 January 2021)	59
5	Video: nearly 100,000 doctors and health professionals unite against COVID-19 “vaccines”	https://lbry.tv/@elinvestigador:0/ask-the-experts-espanol:7 (accessed on 10 January 2021)	37
6	Argentina receives the first doses of Sputnik-V vaccine	https://cnnespanol.cnn.com/video/argentina-vacunacion-sputnik-v-rusia-coronavirus-cnn-primera/amp/?__twitter_impression=true (accessed on 7 January 2021)	36
7	Reluctancy to take the COVID-19 vaccine drops from 47% to 28% in less than 1 month	https://www.eldiario.es/sociedad/reticentes-ponerse-vacuna-covid-19-bajan_1_6521496.html (accessed on 10 January 2021)	34
8	Who is responsible for the eventual health risk? This is why laboratories selling COVID vaccine to Chile are protected from legal liabilities	https://www.latercera.com/la-tercera-pm/noticia/quien-responde-por-el-eventual-riesgo-sanitario-las-razones-por-las-que-laboratorios-que-venden-a-chile-la-vacuna-covid-estan-protegidos-de-responsabilidades-legales/VLSZWS73GVBJ7KVRBWE636DKSI/ (accessed on 7 Januay 2021)	32
9	WHO suspiciously changes the definition of herd immunity	http://euskalnews.com/2020/12/la-oms-cambia-sospechosamente-su-deficion-de-inmunidad-de-rebano-por-que-lo-explicamos/ (accessed on 10 January 2021)	11
10	Encephalitis: the “adverse effect” of the “quick” anti-coronavirus vaccines noted by Dr. Cavadas	https://www.lasprovincias.es/sociedad/salud/pedro-cavadas-alerta-encefalitis-efecto-adverso-vacuna-coronavirus-20201213123502-nt.html#vca=eng-rrss&vcm=amp&vso=lasprovincias&vli=tw (accessed on 10 January 2021)	8

## Data Availability

The data that support the findings of this study are available from the corresponding author upon reasonable request.
